# Trends in the Source of New Enrollees to Medicare Advantage From 2012 to 2019

**DOI:** 10.1001/jamahealthforum.2022.2585

**Published:** 2022-08-12

**Authors:** David J. Meyers, Amal N. Trivedi

**Affiliations:** 1 Department of Health Services, Policy, and Practice, Brown University School of Public Health, Providence, Rhode Island

## Abstract

This cross-sectional study assesses trends in the source of new enrollees to Medicare Advantage plans from 2012 to 2019.

## Introduction

In Medicare Advantage (MA), private insurers receive capitated payments to cover enrollees’ health care needs.^1^ Enrollment in MA doubled from 2011 to 2020, with 42% of Medicare beneficiaries (>26 million people) enrolled in MA plans in 2021.^2^ Recent growth has been greater for Black and Hispanic beneficiaries and those with low income compared with White beneficiaries and those with higher income.^3^

Understanding the source of new enrollment in MA growth may help project future program enrollment and development. From 2006 to 2011, the primary source of new enrollees in MA was those who switched from traditional Medicare (TM) rather than newly eligible Medicare beneficiaries.^4^ The objective of this cross-sectional study was to understand trends in enrollment growth from 2012 to 2019 and characteristics of individuals who newly enrolled in MA or switched from TM.

## Methods

Using the 100% Master Beneficiary Summary File (MBSF) from 2011 to 2019, we identified all beneficiaries newly enrolled in MA annually and classified beneficiaries as newly Medicare eligible or previously enrolled in TM. We characterized differences in demographics between these 2 groups. Race and ethnicity were classified using the Research Triangle Institute race and ethnicity code from the MBSF. Analyses were performed using Stata, version 17. This study followed the STROBE reporting guideline and was determined to be exempt by the Brown University institutional review board, with a waiver of informed consent, because it was secondary research.

## Results

Our study included 524 442 225 person-years from 2011 to 2019. From 2012 to 2019, the proportion of new MA enrollees who switched from TM increased from 65.9% to 71.1% (Figure, A) and the proportion of new enrollees who selected MA increased from 18.1% to 24.7% (Figure, B). From 2013 to 2019, the proportion of returning enrollees who selected MA each year increased from 27.6% to 37% and the proportion of beneficiaries who switched from TM to MA increased from 3.5% to 5.5%.

**Figure.**
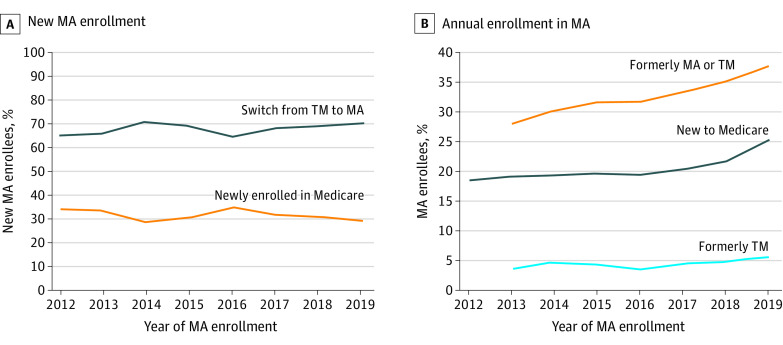
Composition of Medicare Advantage (MA) Beneficiaries Data were from the Master Beneficiary Summary File from 2011 to 2019. A, The denominator for each line is the total number of enrollees who were newly enrolled in MA in a given year. Those who switched from traditional Medicare (TM) to MA were previously enrolled in TM in 1 year and switched to MA in the next year. Those who were newly enrolled in Medicare were beneficiaries who selected MA when they first enrolled in Medicare. B, The denominator for the new to Medicare group was all new Medicare beneficiaries, and the numerator was the number who selected an MA plan. The denominator for the formerly MA or TM group was the number of all previously enrolled MA beneficiaries (regardless of program enrollment), and the numerator was the number who selected MA. The denominator for the formerly TM group was the total number of TM enrollees in the previous year, and the numerator was the number who switched to MA. Data are not presented in 2012 for returning enrollees because 2 years of previous data were required to determine whether enrollees returned.

Compared with MA enrollees who were new to Medicare, beneficiaries who switched from TM to MA in 2019 were older (mean [SD] age, 68.4 [10.9] vs 63.5 [5.3] years), less likely to be Hispanic individuals (10.5% vs 14.6%), and more likely to be Black individuals (14.9% vs 11.9%), to be dually eligible for Medicaid (29.0% vs 19.3%), to have a disability (30.5% vs 16.6%), and to die within 2 years of enrollment (7.2% vs 2.4%) (Table). Between 2012 and 2019, newly enrolled beneficiaries who selected MA were more likely to be Black individuals (11.9% vs 9.4%) and dually eligible for Medicaid (19.3% vs 12.8%). Five years after enrollment, 77.1% of those who switched to MA were still enrolled in MA compared with 85.2% of new enrollees.

**Table.** Characteristics of MA Enrollees by Previous Year Enrollment in 2012 and 2019

Characteristic	MA enrollee^a^						
	2012				2019		
	Already enrolled in MA (n = 11 451 637)^b^	Newly enrolled in Medicare (n = 721 325)^c^	Switched from TM (n = 1 372 964)^d^	Already enrolled in MA (n = 19 736 237)^b^		Newly enrolled in Medicare (n = 934 936)^c^	Switched from TM (n = 2 219 196)^d^
Age, mean (SD), y	73 (10.1)	62.9 (6.0)	68.3 (11.3)	72.8 (10.0)		63.5 (5.3)	68.4 (10.9)
Sex							
Female	6 528 980 (57.0)	410 971 (57.0)	732 163 (53.3)	11 216 835 (56.8)		528 442 (56.5)	1 211 685 (54.6)
Male	4 922 657 (43.0)	310 354 (43.0)	640 801 (46.7)	8 519 402 (43.2)		406 494 (43.5)	1 007 511 (45.4)
Race and ethnicity^e^							
Asian/Pacific Islander	349 610 (3.1)	27 203 (3.8)	53 092 (3.9)	777 966 (3.9)		47 169 (5.1)	79 552 (3.6)
Black	1 195 445 (10.4)	67 646 (9.4)	197 979 (14.4)	2 465 838 (12.5)		111 103 (11.9)	332 068 (14.9)
Hispanic	1 537 602 (13.4)	98 640 (13.7)	179 782 (13.1)	2 818 229 (14.3)		136 711 (14.6)	232 032 (10.5)
Native American/Alaska Native	22 113 (0.2)	1051 (0.2)	3702 (0.3)	40 600 (0.2)		702 (0.1)	7633 (0.3)
White	8 217 493 (71.8)	500 807 (69.4)	918 596 (66.9)	13 203 238 (66.9)		609 851 (65.2)	1 510 408 (68.1)
Other or unknown^f^	129 374 (1.1)	25 978 (3.6)	19 813 (1.4)	430 366 (2.2)		29 400 (3.1)	57 503 (2.6)
Dually eligible for Medicaid	1 905 361 (16.6)	92 426 (12.8)	360 293 (26.2)	4 140 141 (20.9)		180 831 (19.3)	644 490 (29.0)
Reason for entitlement							
Age	9 002 465 (78.6)	578 866 (80.3)	945 579 (68.9)	14 992 517 (75.9)		779 274 (83.4)	1 534 355 (69.1)
Disability	2 430 621 (21.2)	141 479 (19.6)	423 177 (30.8)	4 707 476 (23.9)		154 707 (16.6)	676 625 (30.5)
ESRD	5483 (0.1)	749 (0.1)	1597 (0.1)	15 024 (0.1)		727 (0.1)	4913 (0.2)
Death within 2 y^g^	959 561 (8.4)	16 422 (2.3)	89 560 (6.5)	1 381 477 (8.2)		18 423 (2.4)	124 858 (7.2)
Time after MA enrollment, y^h^							
1	NA	4 981 717 (97.0)	10 068 547 (94.2)	NA		NA	NA
2	NA	4 016 855 (94.2)	7 658 664 (89.3)	NA		NA	NA
3	NA	3 161 525 (91.4)	5 703 380 (84.9)	NA		NA	NA
4	NA	2 404 335 (88.2)	4 266 543 (80.4)	NA		NA	NA
5	NA	1 718 562 (85.2)	2 913 603 (77.1)	NA		NA	NA

Abbreviations: ESRD, end-stage renal disease; MA, Medicare Advantage; NA, not applicable; TM, traditional Medicare.

^a^ Data are presented as number (percentage) of enrollees unless otherwise indicated.

^b^ Beneficiaries already in an MA plan who remained in an MA plan.

^c^ Beneficiaries who selected MA the first time they enrolled in Medicare.

^d^ Beneficiaries who previously enrolled in TM in 1 year and switched to MA the next year.

^e^ Race and ethnicity were classified using the Research Triangle Institute race and ethnicity code from the Master Beneficiary Summary File.

^f^ Other includes categories not captured by Medicare race and ethnicity variables.

^g^ Calculated by checking whether an enrollee had a date of death within 2 years of making an enrollment decision. Data were only available through 2017.

^h^ Calculated conditional on survival and based on whether the enrollee is still enrolled in MA in each subsequent year. The last year of available data was 2019; thus, 1 year after enrollment was calculated for 2012 to 2018 enrollees and 5 years after enrollment was calculated for 2012 to 2015 new enrollees.

## Discussion

This study found that from 2012 to 2019, growth in MA enrollment was primarily attributable to TM beneficiaries who switched to MA rather than to newly eligible Medicare beneficiaries enrolling in MA. Also, beneficiaries who switched from TM were more likely to have a disability or to be dually eligible for Medicaid than were new enrollees.

Several factors may contribute to these findings. Medicare Advantage plans may offer benefits unavailable in TM, such as dental and vision coverage and supplemental benefits for those with chronic illness; often provide lower out-of-pocket costs than TM without supplemental insurance, which prior work has found to be attractive for enrollees^5^; and often have lower premiums than supplemental insurance plans. Supplemental plans may deny coverage on the basis of pre-existing conditions.^6^ Our study is limited in that it was not designed to examine these mechanisms. As MA continues to grow, understanding the reasons for switching from TM to MA will become more important.
